# The Role of Coincidence-Detector Neurons in the Reliability and Precision of Subthreshold Signal Detection in Noise

**DOI:** 10.1371/journal.pone.0056822

**Published:** 2013-02-13

**Authors:** Yueling Chen, Hui Zhang, Hengtong Wang, Lianchun Yu, Yong Chen

**Affiliations:** 1 Institute of Theoretical Physics, Lanzhou University, Lanzhou, China; 2 Department of Physics, Gansu College of Traditional Chinese Medicine, Lanzhou, China; 3 Department of Mathematics, King’s College London, London, United Kingdom; McGill University, Canada

## Abstract

Subthreshold signal detection is an important task for animal survival in complex environments, where noise increases both the external signal response and the spontaneous spiking of neurons. The mechanism by which neurons process the coding of signals is not well understood. Here, we propose that coincidence detection, one of the ways to describe the functionality of a single neural cell, can improve the reliability and the precision of signal detection through detection of presynaptic input synchrony. Using a simplified neuronal network model composed of dozens of integrate-and-fire neurons and a single coincidence-detector neuron, we show how the network reads out the subthreshold noisy signals reliably and precisely. We find suitable pairing parameters, the threshold and the detection time window of the coincidence-detector neuron, that optimize the precision and reliability of the neuron. Furthermore, it is observed that the refractory period induces an oscillation in the spontaneous firing, but the neuron can inhibit this activity and improve the reliability and precision further. In the case of intermediate intrinsic states of the input neuron, the network responds to the input more efficiently. These results present the critical link between spiking synchrony and noisy signal transfer, which is utilized in coincidence detection, resulting in enhancement of temporally sensitive coding scheme.

## Introduction

Neurons transmit information by complex spike sequences that reflect both the intrinsic dynamics and the features of stimulus. However, the coding scheme used in this process is not fully understood, and is often a highly argued issue [1]–[6], which is of critical importance for neuron computation. The traditional view first advocated in [7] considered the cortical neurons as an integrate-and-fire device [1], [2]. In this simple scheme, how neurons encode and process the stable propagation of spiking in cortical neural networks is indicated by their average firing rate [2], [6], which conveys information only with low reliability, and little or no information is thought to be transferred in the timing of the spikes [2]. Furthermore, others suggested that neurons in the cortex work essentially as coincidence detectors [2], [8], [9], and relay preferentially synchronized synaptic inputs and the exact timing of spikes [8], [10], [11], which has been shown to potentially provide information in addition to the spike rate as the coding scheme. Moreover, studies of the visual [9], [12] and the somatosensory [5] systems have shown that neuronal synchronization plays a critical role in sensory information transmission from one brain region to another. Thus, coincidence detection might be a prevailing mode [2], and synchronous inputs are more effective than asynchronously arriving signals. In addition, researchers have argued that coincidence detection is a highly efficient operation mode, and that the cortical neurons are naturally sensitive to coincidence inputs.

Coincidence-detector (CD) neurons have long been studied in a wide variety of central nervous systems. These neurons were advocated in the auditory system [13]–[16], where cells in the binaural cochlear only discharge when receiving coincidence spikes from their afferent inputs. Physiologically, neurons in the medial superior olive are sensitive to their synchronized afferent signals. Such highly specialized neurons can localize sound by using the interaural time difference. The octopus cells of the mammalian cochlear nucleus can detect their synchrony auditory nerve fiber inputs, because of their anatomical and biophysical specialization, and synchrony is relevant to the ability of neurons to encode the temporal features of acoustic stimuli with greater precision, such that they can convey the information more reliably [15]. This improvement in accuracy and reliability is the result of coincidence detection. In addition to the auditory system, it has observed that other mechanisms also depend on coincidence detection, such as the ‘inding problem’in visual system [2], which supports the notion of highly temporal precision [9]. Experimentally, synchronization has been observed in a very large number of regions, from cortical areas to thalamic nuclei, and even in cerebral hemispheres [2]. Thus, coincidence detectors indeed play an important role in neural signaling, and many other neurons can act as coincidence detectors, such as CA1 pyramidal cells [17], which can preserve and even enhance the precise of firing times. Furthermore, experiment on the rat posteromedial barrel subfield have suggested that this field may work as a coincidence detector that processes spatial features.

There are a large number of studies on the properties of CD neurons in information processing [8], [16], [18]–[21]. It is suggested that neural synchronization is a mechanism for facilitating the transmission of sensory information [5], [9] and that correlated spikes strongly increase the probability of firing and ensure the transfer of information from the thalamus to the cortex. Theoretical analyses have also shown that the processing rate of CD neuron is faster than the incoming information rate. Thus, CD neurons are a robust, efficient, and reliable mechanism that decreases the spontaneous rate, and improves the signal-to-noise ratio at the same time [21].

However, previous studies have mostly been performed on only one aspect of single neurons, either integrator devices or coincidence detection. Can these two distinct operations of neurons cooperate together? In the primary work on subthreshold noisy signal detection we found that a single neuron fails, but a simple neuronal circuit can perform detection more precisely and reliably [22]. Here, we explore the issue of the reliable transfer of information in a network model composed of an amount of integrator neurons and one CD neuron. By using the output spikes of Leaky Integrate-and-Fire (LIF) neurons to mimic the synaptic input of the CD neuron, we show how and to what extent the CD neuron can enhance the reliability of weak signal detection. We independently varied two parameters, the threshold and the detection time window, which determine the intrinsic states of the CD neuron, to study their roles in the control of the precision and the reliability of the response of the network. Our results show that, the correlated spikes from multiple presynaptic neurons strongly increase the firing probability of postsynaptic neurons [23] and ensure the transfer of signals when the signal is synthesized by the CD neuron. These results are consistent with previous theories [9], [23], [24]. Moreover, the reliability of the spike output can be predicted using the threshold of the CD neuron, which is the number of presynaptic inputs required to activate the CD neuron and is driven by the same input trains within an optimal time-window that varies in a larger range.

## Methods

### Model formulation

It has been shown that the synchronous firing of groups of neurons is a mechanism by which sensory information is processed [25]–[27]. It is possible for information to propagate stably if the number of firing neurons in a small time window is sufficiently large and the initial variability is sufficiently small.

**Figure 1:**
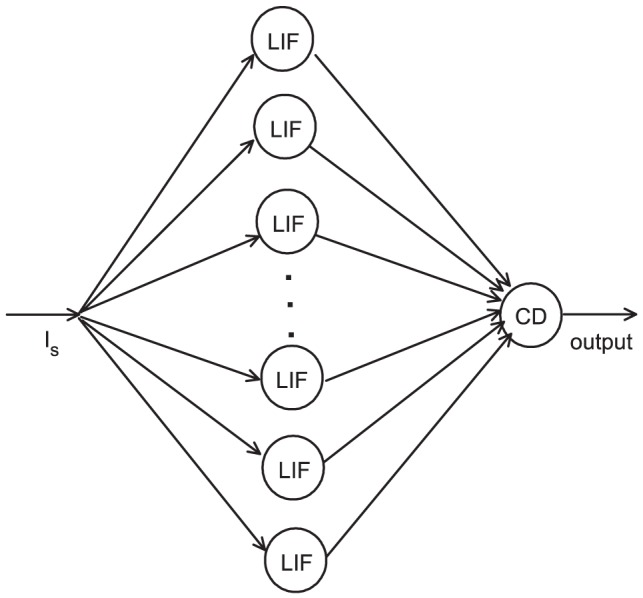
Sketch of the neural network model. 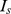
 is the subthreshold input pulse. There are 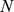 independent LIF neurons with Gaussian white noise stimulated simultaneously. Each LIF neuron generates a spike randomly and projects it to a targeted the CD neuron. If the input spikes of CD neuron achieve the threshold 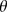 within time window 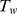, it fires one spike.

Here we investigate the enhancement of the precision and the reliability of spikes at a single CD neuron that receives convergent input as its presynaptic signals. We construct a simplified neural network model to study the properties of the CD neuron (see Figure 1). The neuron receives the input train from dozens of LIF neurons with Gaussian white noise that are stimulated simultaneously. The LIF neuron is a point neuron [28], [29], neglecting the special dendritic structure of the neuron, because we do not consider the dendritic processing in those cells. The CD neuron fires one spike for the first time when its input spikes exceed the threshold 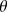 during a proceeding time window 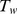. Additionally, the output of the network is emitted at the moment of the last input spike. The stimulation 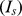 is subthreshold for LIF neurons, but each neuron can fire a spike randomly under the noise. The firing probability of these neurons can be solved using the proper approximate method (for details see [29]).

#### LIF neuron model

The LIF model is simple, but it is sufficiently complex to capture the information processing capabilities of neurons. It usually describes the subthreshold properties of the membrane voltage, whereas the generation of action potential is generally not considered as an intrinsic part of the model. The membrane potential 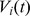 of each LIF neuron is described by the following differential equation [28]

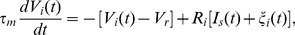
(1)


where 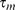 and 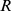 are the membrane time constant and the resistance of the neuron, respectively, and they are identical for all LIF neurons. 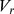 is the rest potential, and 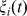 is the Gaussian white noisy due to background synaptic input, with zero mean 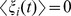 and autocorrelation 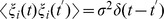. When 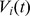 reaches the threshold 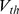, the neuron sends out an action potential or a spike to the connected CD neuron and resets its membrane potential to the reset potential 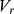. We choose an absolute refractory period for all LIF neurons, 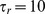 ms. In the simulation, a refractory period 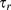 of CD neuron is also defined by 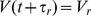. For convenience, the initial potential is set to the rest potential, 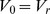.

The current 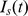 is given by
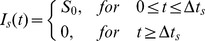
(2)


where 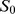 is the pulse intensity and 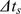 is its stimuli duration. For a small duration, the intensity must be sufficiently large to cause the neuron firing at least once, while for small input, a longer duration is needed. The distribution of the first passage time can be observed using simulations by applying a current pulse repeatedly to a neuron and accumulating the firing times into a peristimulus time histogram (PSTH) [30], [31].

#### CD neuron model

Coincidence detection is one way to describe the functionality of a single neuron. If the integration interval, over which neurons summate the presynaptic inputs effectively, is short compared with the interspike interval, neurons act as coincidence detectors [2]. CD cells are frequently found in different neural structure [32]. Experimentally, in the auditory system the medial olive (MSO) and the lateral superior olive (LSO), the first nuclei in the sound localization pathway, are the highest spike precision in mammalian central nervous systems [15]. To achieve sufficient time precision, the coincidence detection mechanism must be used to compute the direction of sound [15], [33]. CD neurons have been studied in other different levels of the auditory system [34]–[38]. Moreover, CD neurons have also been investigated in neocortical pyramidal neurons [17], [39], [40] in experiments and theories. The CD neuron receives converging synaptic inputs, where each input is subthreshold. The neuron will not fire unless these inputs are temporally sufficiently close together. That is to say, the synchronous arrival of the synaptic inputs may push the membrane potential of the CD neuron over the threshold and generate an output spike.

The aim of the present work is to determine directly the reliability of weak signals response and temporal precision with which the CD neurons are capable encoding a stimulus into a spike train. A simplified logical CD neuron model is introduced to characterize directly the main properties of the CD neuron to convert a temporal code into a rate code. The presynaptic potential is generalized by multiply LIF neurons, which are simple but are sufficient for our work. Here, we are not concerned with the mechanism of action potential both for presynaptic and postsynaptic neuron. As a result, we didn't care about here what the intrinsic mechanism for the CD neuron to realize the functions of the integration and synchronization. We just use two parameters, the size of detection windows (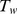) and the number of synchronous input spikes 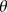, to describe the properties of CD neurons. In this work, we manly focus on whether the task of subthreshold signals detection can be done by a simple neural network.

The issue of how coincidence detection can be performed and what is the modulate mechanism have been discussed by several groups [41]–[43]. In this article, we found that there exits an optimal threshold of 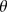 for the CD neuron with best performance. Although the CD neuron model in our work is simplified, it is the same as the other models such as Hodgkin-Huxley (HH) and LIF neuron model in calculating the output firing rate of a single neuron. For example, a CD neuron could be realized by a LIF or HH neuron with a very small value of 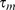 so that the neuron would be able to integrate effectively only the spike arriving at a temporal window 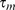 (which here would play the role of 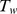, as we will describe in the following). For the HH neuron model the value of the threshold is fixed and cannot be varied. Therefore we can vary the strength of the presynaptic inputs in the simulation. This thresholds have the similar meaning in our CD neuron model.

In this work, each neural input is represented by sets of spikes that occur at instances and are generated by 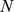 independent LIF neurons (Figure 1). If the mean firing probability of each LIF neuron during time 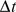 is 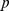, then for the independent inputs with a mean rate of 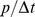, the probability of receiving at least 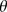 synchronous input spikes for the CD neuron to produce an output spike is [22], [44]

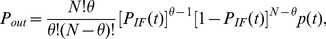
(3)


where 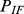 is the cumulative distribution function of the firing probability of the individual LIF neuron, 
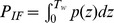
. 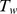 is the detection window of the CD neuron and 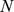 is the total number of LIF neurons.

Note that Equation (3) is an idealization of biological firing because the action potential depends on the recent history of cell firing but there is no refractoriness in this equation. After firing a spike, it may be virtually impossible to initiate another spike, which is called absolute refractory period. In our simulation, we set the firing rate of CD neuron to zero immediately after a spike is triggered.

### Analysis

#### Post stimulus time histogram

In our simulation, the post stimulus time histogram (PSTH) is measured as follows. The rectangular current pulse is in the form of Equation (2) at time 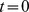 with a width size 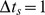 ms, and the time duration of every trials is 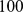 ms. Thus, the firing probability of the neuron under 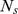 stimuli is given by
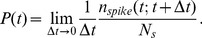
(4)


Here, 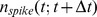 is the number of spikes emitted between 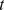 and 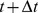.

#### Interspike interval distance

A spike train can be defined as sum of 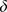 functions
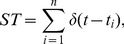
(5)


where 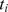 denotes the initial time of the 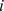
th pulse and 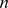 is the number of pulses. Accordingly, 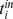 and 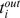 are the input and output spike times of the network, respectively.

Then, for a input train, the interspike interval (ISI) can be calculated with [45]

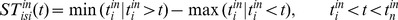
(6)


Similarly, one can calculate 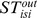 for the output train. The synchronous measure between two spike trains is
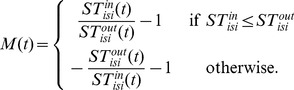
(7)



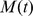
 becomes zero when the input and output trains have the same frequency but approaches to 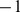 or 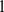 if the CD cannot respond properly or more than once for one input. Using the spike-weighted normalization, we obtain
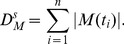
(8)


Actually, this method has been widely used to quantify the similarity between the spike trains from the recording of cortical cells in vitro [45]. Furthermore, it does not involve any parameters and is self-adaptive.

## Results

### The firing-rate response of the CD neuron

To examine the temporal properties of the CD neuron, we find the firing probability of the CD neuron obtained from the PSTH. Here, for simplicity, the CD neuron works as a logical judgment device, and its convergent presynaptic inputs are independent. We extract the PSTHs from 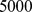 trials (time duration is 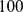 ms) with a bin size of 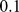 ms. At time 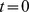, a rectangular current pulse is injected.

**Figure 2:**
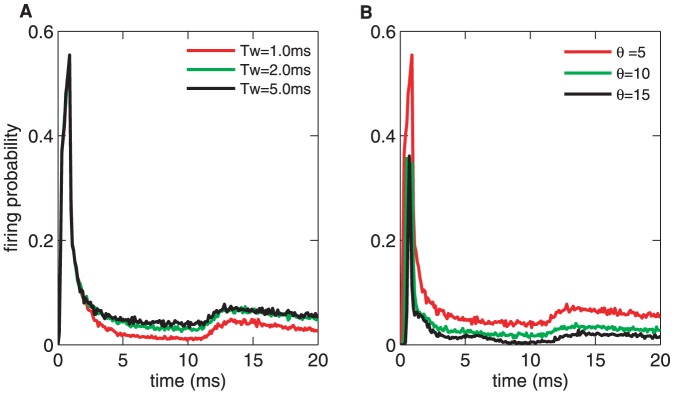
PSTH of the CD neuron. The neuron network responds to a rectangular current for (A) various 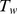 values with fixed 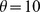 and (B) various thresholds 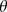 with the time window 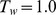 ms. In all cases, the input on the LIF neurons in the first layer is below the threshold 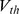 and the embedded noisy current input is Gaussian white noise with a noise intensity 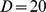 mV/ms. The PSTH is obtained from 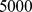 trials. Simulation parameters: the LIF neruon threshold, the resting potential and the membrane time constant are 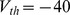 mV, 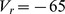 mV and 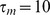 ms respectively; the number of LIF neuron is 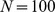; the time step 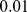 ms.

As shown in Figure 2, when the current transient occurs, the neuron discharge increases sharply from its spontaneous firing rate until reaching the maximal firing rate, the peak of the PSTH. Then there is a rapid decrease in the firing probability and the duration of the response restraint is approximately 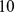 ms, due to the refractory period of its presynaptic LIF neurons.

To evaluate the role of the time window and threshold on the detection of the presynaptic spike synchrony, we compare the spike responses elicited by the CD neuron with a constant 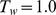 ms and a variable 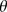 (Figure 2A) or, alternatively, a variable 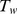 and a constant 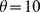 (Figure 2B). In all cases, the synaptic inputs are independent and the number of LIF neuron is 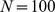. Figure 2A depicts the firing probability of the CD neuron with three detection time windows (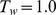, 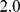, 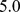 ms) for responses evolved with rectangular subthreshold stimuli. The PSTH for smaller 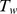 has a lower baseline than those for a larger time window, which indicates that the narrower 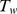 inhibits the spontaneous firing rate (the baseline of the PSTH) but has no effect on the firing rate (the peak of the PSTH). However,the effect of a larger 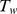 on the firing rate is weaker. For a threshold (see 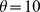), there is a critical 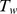 value after which it no longer influences the shape of the PSTH anymore. In the following text, we will observe that for a given firing threshold, there is an optimal detection time window, and, in this state, the neuron can detect the input signals with both high precision and high reliability.

Figure 2B presents the PSTHs for different 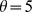, 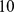 and 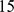 with a fixed 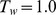 ms. The PSTH for a larger threshold has a lower peak and a lower spontaneous firing rate than smaller thresholds. Moreover, the width of the peak tends to narrow with increasing 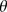, which means that information is encoded more accurately for a larger threshold than for a lower one. Thus this network is able to achieve a high firing rate (the high peak in PSTH) and a low spontaneous firing rate(the low baseline of PSTH) in response to stimuli with appropriate parameters. In other words, it is possible for the CD neuron to facilitate more reliable neuronal operation by improving the firing rate and depressing spontaneous spikes [21].

It is well known that spike timing can react the presence of information [26]. Normally, two measures, *reliability* and *precision*, are extracted from PSTHs [46]. Reliability is defined as the fraction of the total spikes during the first peak of the PSTH, and precision is the standard deviation of this peak. By manipulating the detection time window 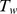 and computing the reliability or precision, one can identify the optimal firing threshold when the information is best encoded. For example, the optimal 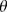 for a given 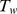, or alternatively, the optimal 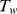 for a given 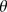 can be identified. That is to say, there exists a appropriate pair of 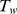 and 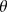 that provide the optimal encoding of the input information.

**Figure 3:**
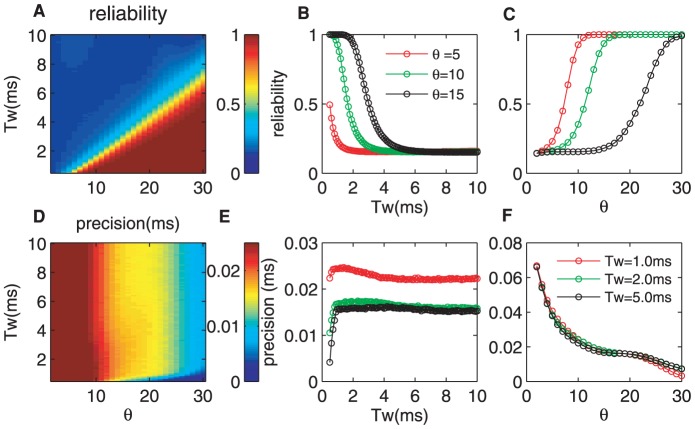
Influences of the detection time window 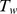
 **and the threshold** 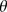 **of the CD neuron on the detection reliability and precision.** (A) The reliability as a function of 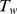 and 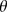. Note that there exits a clear boundary for high and low reliability. Almost 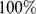 detection reliability can be achieved for proper pairing of 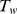 and 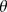. (B, C) Detailed views of parts of (A) with different 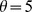, 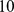, 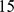 and 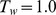, 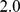, 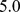 ms, respectively. (D) The detection precision as a function of 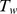 and 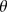. (E, F) Detailed views of parts of (D). It is clear that 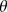 has a significant effect on the precision for any time windows. The parameters are 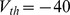 mV, 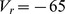 mV, 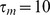 ms, and 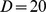 mV/ms.

Figure 3A plots the reliability as a function of the threshold 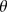 and the detection time window 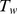. There exists a regime (warm color, the bottom right in Figure 3A) in which the reliability reaches the maximal value, and is approximately equal to one. The reliability is enlarged over a wide parameter range, which indicates that the CD neuron enhances the signal reliability as a general rule. However, this enhancement is not always efficient, and there exists a boundary. That is to say, although the reliability reaches approximately one within the warm-color regime, the encoding is not efficient for the neuron. For example, when 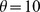, 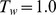 ms is sufficiently large for the CD neuron to encode all of the information reliably, and increasing 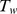 will not improve the reliability. As shown in Figure 2A, for values larger than the optimal pair of values of 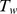 and 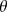, the firing rate does not change with 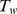. Thus, when 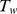 increases, its restriction has less effect, and the reliability attains the best value on the boundary.

Figure 3B illustrates the reliability versus the detection time window 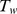 for 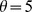, 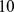 and 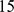 to demonstrate the role of 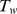 in detail. For small 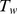, the delay of the response is not large enough for the neuron to fire spikes for all inputs, and its spontaneous activity is extremely inhibited (a zero baseline and low peak in the PSTHs, see Figure 2A). Therefore, the reliability has a maximal value for any firing thresholds. For example, the reliability is equal to 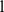 for 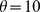, but it is 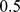 for 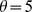. It is clear that the maximal reliability will be very small if 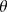 is too small because a much smaller firing threshold cannot inhibit the spontaneous firing and may even have no effect on this activity. That is to say, although the peak of the firing rate is very high (Figure 2C, 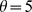), the reliability of the neuron response does not reach its optimal value. There must be a proper 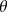 for which the neuron best transfers information for any 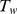. In Figure 3A, it is shown that the reliability may reach the optimal value, 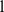, for 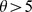. When 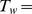
1 ms, 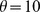 is sufficient for the neuron to fire best, similarly for 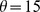 (see Figure 3B). However, in terms of the coding efficiency, using 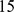 or more synchronous synaptic inputs is a waste. In contrast, when 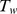 is much larger (for example, 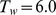 ms), the neuron has fired all of its spikes. The larger time window neither induces the neuron to fire more nor increases the spontaneous firing. Thus, the reliability decreases and has a much smaller value for any thresholds.

Figure 3C plots the relation between the reliability and 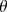 for 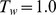, 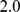 and 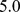 ms. The reliability is enhanced with increasing 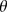 and tends to 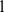. For example, 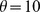 can transfer the information with a reliability close to 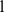, and the spontaneous firing is completely inhibited. With further increases in 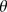, the reliability remains at the same value, and only the firing rate and the peak of the PSTHs are reduced. Thus, Figure 3C shows a sigmoidal function curve with 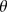, and the slope is determined by the degree of the input synchrony, 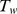.

The results above demonstrate the following significant findings on the reliability of coincidence detection for excitatory synchronous input. First, there exists a sharp transition in reliability for both 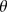 and 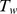. By choosing 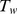 and 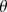 properly, it is possible to maintain a the reliability of 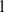 (or the detection reliability is 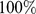). Second, neurons with too high a threshold or too small a time window cannot fire evenly. However, for too low a threshold or too large a time window, the reliability is close to zero because the spike firing is overwhelmed by spontaneous firing activity. Finally, for a given detection window, there is an optimal threshold at which neurons have the maximal reliability, and a larger threshold is unnecessary. Similarly, for a given threshold, there exists an optimal detection time window, and a larger size of window has no effect. The neuron is not required to wait a longer time before firing.

Next, we focus on how the intrinsic state of the CD neuron influences the detection precision. Figure 3D shows the detection precision versus 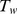 and 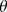. The output of the CD neuron becomes more accurate as 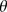 increases. For moderate 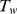 and 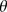, the peak width of the PSTHs becomes very narrow, and the baseline is zero (Figure 2). This improvement in the detection precision increase when 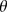 is somewhat larger than 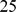 (the blue region in Figure 3D).

To show the improvement in precision in detail, we vary 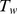 (Figure 3E) or 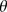 (Figure 3F). The neuron with a small 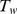 but a large 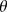 processes the input information more precisely, for example 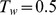 ms and 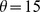 in Figure 3E. The precision decreases with 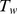, when it is not too large. Indeed, the larger detection time windows easily lead to spontaneous firing. However, a much larger 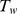 fails to decrease the precision because the firing has been saturated. In addition, the precision increases with 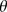 in all ranges of 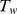 considered (Figure 3F). Although a higher threshold increase the accuracy, when 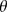 is too high,it will reduce the regular firing rate. Thus, only the CD neuron at an intermediate state can encode the inputs with highest precision. In addition, there even exits fully synchronized status. Therefore,the dependence of precision on 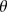 reflects the output spread, but the threshold can also represent the precision, which leads to a higher temporal precision for larger threshold. Moreover, there is a point of intersection in the precision-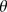 curves for different 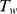 at approximately 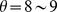. These results suggest that the spike time can be sufficiently accurate by combining 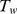 and 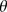 properly.

As we discussed above, there exists a boundary between high and low reliability/precision. By combining Figure 3A and D, it is clear that the reliability can reach almost 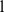 and the precision can be raised to 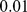 ms simultaneously for a higher threshold and a narrower time window. Moreover, the correlation between the detection time window and the precision is weak, but the correlation is strong for 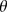. However, there exists a strong correlation between the reliability and 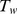 as well as 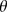, which indicates that the effect of the input synchrony might not be related to the time distribution of synaptic inputs.

Coincidence detection is an efficient pattern [2], [3], [5] where a single neuron can determine the essential state properly. The intrinsic properties of CD neuron depict the magnitude and intensity of synaptic synchrony. CD neurons are more sensitive to the input synchrony. In a word, CD neurons significantly enhance reliability and precision.

### Effect of the refractory period

**Figure 4:**
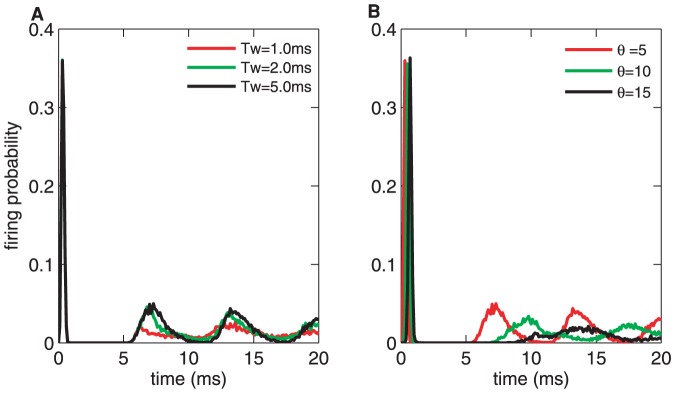
Influences of the refractory period on the PSTH of the CD neuron. The spontaneous firing rate exhibits a highly oscillation but it can be suppressed by the suitable intrinsic properties of the CD neuron. (A) The PSTHs of the CD neuron for 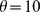 and 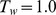, 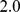 and 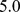 ms. The first peak width of the PSTH becomes narrower and narrower with decreasing 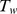, and the fluctuation of the baseline disappears when 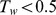 ms. (B) The PSTHs for 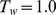 ms and varying 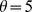, 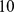 and 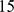. With increasing 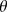, the time delay of the first peak of the PSTH becomes longer. When 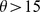, the CD neuron depresses the oscillation. The PSTHs are obtained from 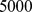 trials with time steps of 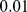 ms averaged to 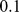 ms. The refractory period of the CD neuron is 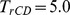 ms. The other parameters are, 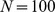, 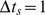 ms, 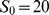 mV/ms, 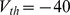 mV, 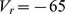 mV, and 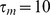 ms, 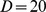 mV/ms.

The refractory period is an intrinsic neuronal psychological phenomenon. The firing probability of a neuron relies stringently on the history of previous spikes [47]. The average firing rate and the instantaneous firing rate are deeply affected by the refractory period. For example, the spontaneous firing rate exhibits oscillation in Figure 4. Especially in the cochlear afferent the refractory period was found to inhibit the firing rate relative to the free firing rate [48].

Figure 4 plots the PSTHs of a CD neuron with a refractory period of 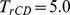 ms. With the presence of the refractory period, the firing probability shows a subtle but noticeable change in the peak and baseline of the PSTH. Compared with the free state (the case of no refractory), the firing rate decreases as reflected by the lower peak in the PSTH. Moreover, there are some periods in which the spontaneous firing rate is close to zero in repeated trials due to the refractory period. During such periodic oscillations, the firing probability increases from zero to a local maximum and then falls back to zero. In some cases, the spontaneous activity clearly becomes jitter, which is modulated by the free spontaneous firing activities. However, with a moderate threshold 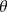 or 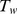, the neuron fires without oscillation. For small 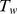, the CD neuron fires with high probability, yielding a PSTH with a narrow peak width and a low oscillating baseline (see Figure 4A). For larger 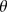, the firing rate shows the same changes, including a small peak width and a low baseline oscillation (Figure 4B).

Moreover, the peak width of the PSTH is actually smaller than that for no refractory period. In Figure 2B, we illustrate the PSTHs of the CD neuron without refractory period for different 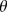  =  5, 10 and 15 with a fixed 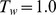 ms. With increasing 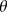, the peak width of the PSTH shrinks. In Eq. (3), the firing probability density function of the CD neuron is given by the order statistics, which is derived from the joint density of the firing probability of the LIF neurons. When 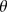 increases, the number of the probability function of input signals increases. As a result, the joint density decreases. So the peak value of PSTH decreases and its width shrinks.

Comparing Figure 4A with Figure 2A, the peaks of the PSTHs exhibit some similar changes in the case of increasing 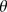 that the peak reduces and the width shrinks. Considering the refractory period of the CD neuron, the firing probability of CD neurons can be calculated as a joint density function, 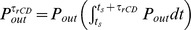. Here, 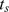 and 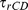 are the last firing time and the refractory period of the CD neuron respectively. In the simulation, 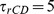 ms. So the peak reduces and its width narrows. Thus we concluded that, in the present of refractory period 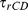, the effective firing threshold is enhanced and the performance of CD neuron is more efficient.

The refractory period leads to a leftward shift in the firing rate curve (see Figures 4A and 2A). The peak value of the initial firing rate of the CD neuron is reduced sharply by the refractory in the case of a smaller firing threshold. For example, when 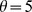 (the red curves in Figures 2B and 4B) the peak values changes from 0.6 to 0.4. Thus 0.4 is the maximal firing probability for the CD neuron with refractory, and the neuron doesn't need a longer time to fire more spikes, because it has already fired all the spikes and the refractory period inhibited the next firing. Consequently, the firing variability of the CD neuron is reduced due to the refractory period.

For some detection time window, increasing firing threshold leads to a rightward shift in the firing rate curve (see Figures 4B and 2B). This is because, for larger 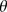 the CD neuron requires more time to reach its firing threshold. The refractory period facilitate this rightward shift since it depresses the next firing and improves the effective firing threshold of the CD neuron.

**Figure 5:**
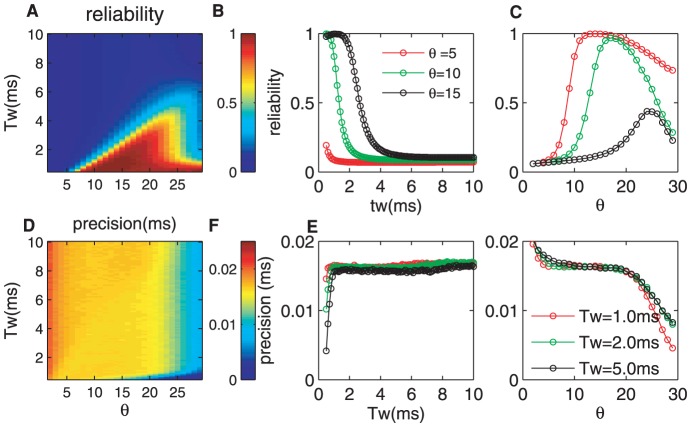
Influence of the refractory period on the detection reliability and precision. (A) The reliability as a function of 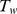 and 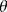. (B, C) 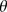 and 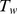 dependence of the detection reliability respectively. (D) The detection precision as a function of 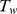 and 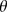. (E, F) 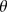 and 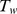 dependence of the precision respectively. The refractory period 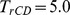 ms. The parameters for the LIF neuron are 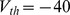 mV, 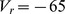 mV and 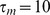 ms. Other parameters are 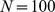 and 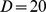 mV/ms.

It is well-known that the refractory periods enable a low spike-count variability at moderate firing rates [24], [49]. In Figure 5A, we plot the reliability of the network output in terms of the refractory period. The improvement in the reliability is demonstrated when the firing thresholds are somewhat high (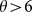) because the refractory period of the CD neuron inhibits the high spontaneous firing (see Figure 4). The high reliability is also restricted to a small detection time windows, 0.1 ms 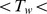 3.0 ms (warm color). When 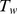 is outside of this range, the reliability decreases dramatically (for example 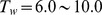 ms, from the red range to the blue range), and the signal detection (in the blue range) is difficult due to high spontaneous firing and jitter (Figure 4). Further, the reliability decreases with 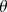 after the firing threshold exceeds its optimal value (at which CD neuron has the optimal reliability), instead of increasing as in the free state (see Figure 4A).

Furthermore, we illustrate the reliability versus 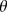 for 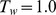, 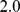, and 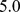 ms, and versus 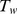 for 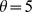, 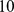 and 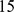 in Figures 5B and C, respectively. The dependence of the reliability-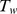 curve (Figure 5 B) is similar to that for the free state (Figure 4B). However, for a slightly low threshold such as 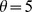 (red curve), the reliability decreases quickly from 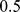 to 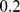 (Figure 5B). Even in this situation, the optimal reliability can be reached with a proper pairing of 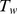 and 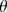. In addition, the relation between the reliability and 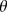 clearly changes. There exists a peak in each curve at which the reliability reaches the maximal value which is much smaller than that of the free state. Thus, the refractory period of the CD neuron narrows the high reliability region compared with Figure 3A. Therefore, although the refractory period reduces the firing probability, it is still possible to transfer information correctly.

The numerical analysis indicated that the absolute refractory period serves to improve the response precision and increases the information transmission rate [47]. Our simulation results shown in Figure 5(D-F), are in agreement with these studies. In Figure 5D, the high precision scope is larger than that for no refractory period. Thus, the refractory period of the CD neuron improves the detection precision (narrows the peak width in Figure 4) of the response to the afferent inputs. This result is consistent with the phenomenon observed in the auditory system where the refractoriness reduces the variance of the cochlear afferent to vowel sounds from the corresponding value with no refractoriness [48]. Furthermore, if we preset the precision, for example, a precision of 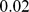 ms, the number of the required synchronous synaptic inputs is reduced due to the refractory, from 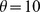 to 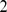. Thus, the refractory period improves the efficiency of neuron coding. Thus, for different stimulus patterns, the CD neuron can perform the subthreshold signal detection reliably and precisely by adjusting its internal status appropriately. Although the refractoriness induces the fluctuations in the spontaneous activity, it is conjectured that the effect of the refractory period can be suppressed by selecting the intrinsic parameters of the CD neuron. That is to say, CD neurons essentially inhibit the oscillation caused by the refractory period and improve the detection of neural signals [47].

### Pulse detection

Synchronization has been proposed to allow neurons to communicate and cooperate with each other and may plays an important role in binding problem [50], therefore making information transfer between neurons reliable. For many years, investigators have shown that if the number of presynaptic inputs is sufficiently large and the spike timing deviation between neurons is sufficiently small, reliable propagation can be achieved.

To illustrate visually the ability of the CD neuron in subthreshold signal detection, we calculate the relative timing of input and output trains, by using the ISI-distance (for a description of the method see [45] or the analysis section) to study whether the timing of responses of CD neuron is locked to the timing of the stimulus and whether the locking degree depends on the intrinsic states of the CD neuron. The ISI-distance is defined as a quantity representing the following properties of CD neurons. The state with zero ISI-distance for a particular stimulus allows the neuron to accurately read out the timing of the input. As described above, by manipulating the parameters of the intrinsic status of the CD neuron, one can identify the optimal detection time window in which information is best encoded. For example, there exists an optimal 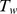 for a given threshold 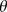. Alternatively, the optimal threshold can be achieved for a given 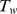.

**Figure 6:**
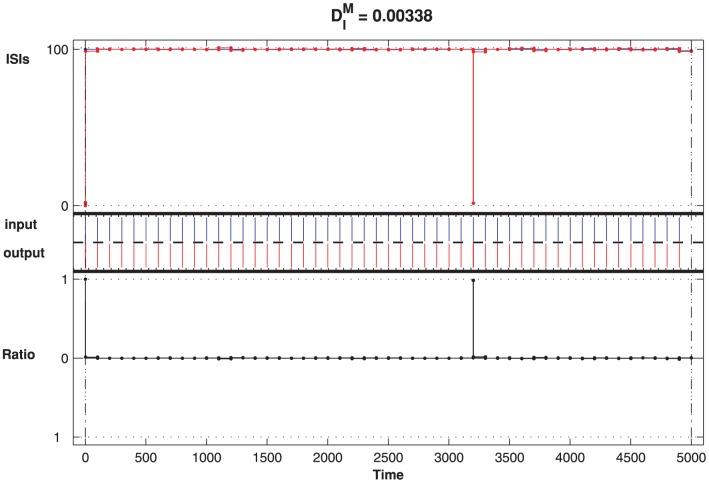
CD neuron with modulated state performs one to one locking. The detected output spikes are marked in red and the blue is the input. The top panel depicts the ISI-distance and the bottom one is the corresponding renormalized ISI-distance. For this pair of spike trains, 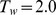 ms and 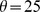, and the ISI-distance is 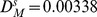. The other parameters for the input and the LIF neurons are the same as in Figure 3.

**Figure 7:**
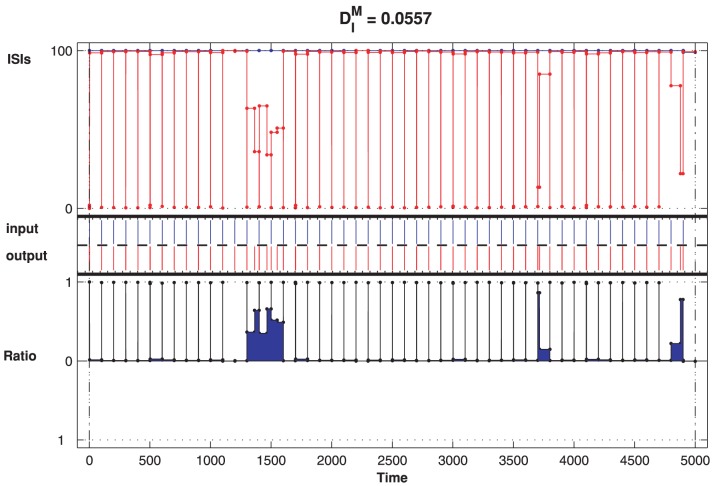
CD neuron fails to respond to the network input. For 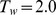 ms and 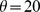, the blue color denotes that the CD neuron cannot detect the synchrony of its inputs properly and the response is faster.

In Figure 6, the ISI-distance of the CD neuron is calculated for input-output spike trains with a 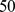 s duration in the case of 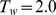 ms and 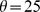 for CD neuron. The middle panel of Figure 6 plots the input spike trains (blue) and the output spike trains (red). The top and bottom panels depict the normalized ISI-distances where 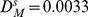 and the input and output spike trains are 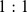 synchronized. This synchronization indicates that the CD neuron can detect the network input precisely under the given parameters. However, in other cases, there is some deviation and the outputs no longer follow the input but are faster (the positive ISI-ratio marked with blue color in Figure 7). Obviously, the spontaneous firing of the network due to the noise cannot be suppressed by the CD neuron, and the neuron is not in the optimal detection states.

**Figure 8:**
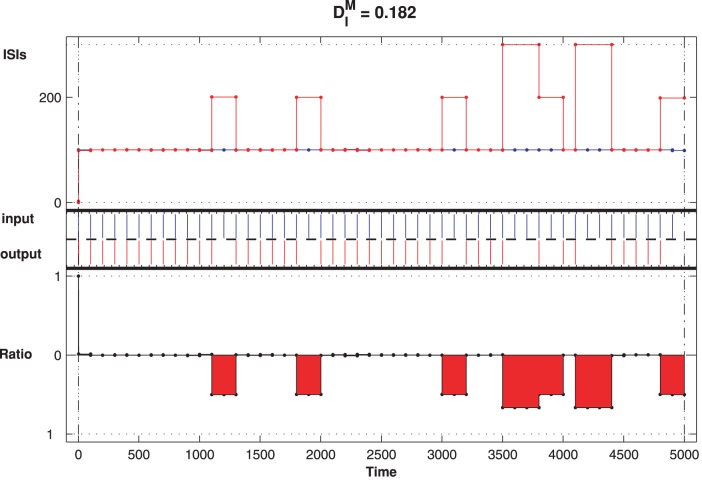
CD neuron fails to follow the network input. For 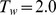 ms and 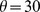, the red color denotes that CD neuron cannot detect the synchrony of its inputs properly but is slower than the input of the network.

However, this does not mean that the higher the threshold is, the less is the normalized synchronous measurement 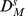. Figure 8 illustrates the example of a much higher firing threshold. The output is slower than the input, and the neuron no longer responds properly to the input. The primary reason is that the CD neuron with a higher threshold depresses not only the spontaneous firing but also the regular firing. In the case of 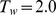 ms and 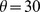, the reliability equals 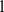 (the Warm-colored regime in Figure 3A). Thus, the optimal pairing parameters correspond to the cases where the neuron inhibits the spontaneous activity completely. These parameters are located on the boundary of the red color in Figure 3A.

Furthermore, for the CD neuron with a refractory periods of 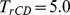 ms, the locking phenomenon emerges for smaller thresholds (figures are not shown at here). We suggest that the CD neuron improve the time precision of the network and that the refractoriness further enhances the neuron's efficiency by decreasing the firing threshold. Moreover, coincidence detection can inhibit the spontaneous firing induced by the intrinsic noise of the network and detect the subthreshold signals precisely. This process is performed by moderating the intrinsic states of the neuron itself.

**Figure 9:**
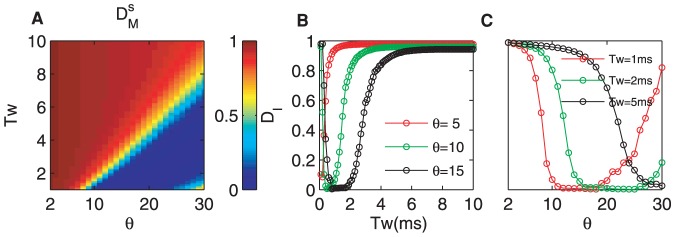
ISI-distance as a function of the time windows 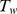
 **and the threshold** 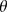 **of the CD neuron under the pulse input.** (A) 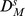 as a function of 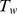 and 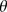. There exists a clear boundary for large and small 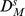, and it can reach approximately 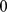 for appropriate pairing of 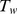 and 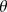. (B, C) 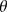 and 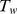 dependence of 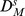, respectively. The parameters are 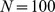, 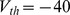 mV, 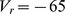 mV, 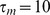 ms, and 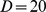 mV/ms.

To examine the synchrony between the input and output trains as a function of 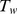 and 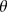, we investigate the behavior of 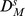 with a number of presynaptic inputs of 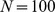. In Figure 9A, as 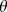 increases, 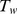 for the small ISI-distance increases significantly, and the range of 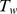 becomes wider and wider. In the case of values that are too large or small for 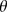, the ISI-distance is large. The normalized synchronous measurement reaches the optimal value, approximately zero, in the black blue range in the figure.

To understand this phenomenon in detail, we plot the 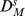 as a function of 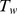 or 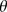. When 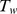 is very small but 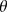 is high (see 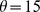 and 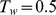 ms in Figure 9B), 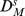 drops significantly to almost zero. However, when 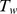 is much larger, the responses of the CD neuron are saturated for all firing thresholds (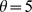, 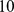, and 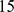). In the range of higher 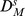 (Figure 9A), the spontaneous activity is too strong for the neuron to detect the signals properly, or the firing threshold is too high for it to fire a spike following each pulse input. Figure 9C presents the 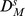 as a function of 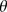. As 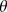 increases, 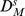 drops to zero. However, 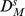 increases with further increase in 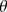, which indicates that the neuron has to be in an intermediate state to response reliably and precisely.

## Discussion

The synchronization of excitatory synaptic inputs onto a target neuron leads to a higher firing rate [51], [52]. However, how this synchronous behavior can be read out is not fully understood. In this paper, we investigate how the CD neuron enhances the reliability and precision in detecting subthreshold noisy signals. The results show that the CD neuron can reliably detect the synchronous spike arriving from different afferent spikes. We also determine the ranges of parameters that defined the neural dynamics of a CD neuron within which the performance of the network model is improved.

We examined the modification of the CD neuron for presynaptic inputs elicited with different degrees of synchrony by independent Integrate-and-Fire neurons. The functional consequence of this modification is analyzed for the precision and reliability in information processing by varying the intrinsic states of the CD neuron defined by three indices: the threshold 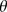, the detection time window 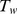 and the refractory period. It is shown that the CD neuron enhance the accuracy of firing times, which is related to the intrinsic properties of the neuron. We interpret the occurrence of a narrow time window and a high threshold (high precision and reliability Figure 4) as evidence of synchronous excitatory inputs. It is conjectured that the CD neuron can predict the synchrony level of presynaptic inputs, and coincidence detection is more reliable than the temporal integration. It was hypothesized that coincidence detection can serve as a mechanisms to bind distributed neuronal activity. For example, in CA1 pyramidal neurons could combined the spatial and temporal context and act as coincidence detectors [17]. Thus, the coincidence detector neuron plays a very important role in reading out and transferring information that may be encoded by synchrony of excitatory inputs.

Computational studies of searching over information transmission have discovered some parameter sets that facilitate the information processing, highlighting the existence of optimal firing threshold for CD neuron [20], [41]–[43]. Pantic *et al.* investigated, analytically and numerically, different types of coincidence detection tasks containing both the coincidence detection of presynaptic cells, synchrony bursts, as well as the synchronous increase of firing rate. They showed that with synaptic depression there exits a range of threshold values which enable the coincidence detection over the large range of input frequencies.

Here we showed that CD neurons can process the subthreshold signals with high precision and reliability by modulating its intrinsic states, but we didn't present what is the modulation mechanism due to the simplified model of CD neuron. We also reported that there is an optimal threshold for the CD neuron to transform information at best. This is consistent with Ref. [42]. The only difference is that we use the detection time window (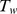) instead of the frequency in [42]. Clearly, these two parameters play the same roles. Indeed, increasing 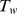 of the CD neuron means more spikes arrive within the window, and it is the same as if we just increased the presynaptic frequency. In addition, though we use the more simplified model to illustrate the main properties of the CD neuron, the less parameters is benefit to do analysis more directly. The presynaptic inputs in our model is realized by multiple LIF neurons, which is more realistic for biological neurons. We believe that our results are solid and it is confirmed further that the coincidence-detection ability of the neuron is independent on the neuron model [42].

Other studies [20], [41], [43] also showed in theory and simulation that there is an optimal threshold for the neuron to detect the temporal correlations between different presynaptic neurons. It is shown that the synaptic facilitation mechanism enhances the coincidence detection of the neurons compared with the case of only depressing synapse [43]. In [41], the authors demonstrated that the coincidence detection properties of the IF neuron depend on the location of the threshold relative to the mean voltage. And there exists an optimal threshold for coincidence detection, at which both spike rate and the coherence gain are high. Moreover they showed that shorter neural time constant yields better coincidence detectors for each of the optimal threshold.

Our findings not only confirm these previous results but also illustrate that when the neuron is at the optimal threshold it can read out the subthreshod signals with high reliability and precision. Moreover, the neuron has potentially the chance to adapt itself to the optimal states for best coincidence detection [41], [42].

Furthermore, the optimal threshold is a general rule for the CD neurons to read out the noisy signals and it is not relevant to the specific modulation mechanisms due to this simplified neuron model. We believe that these conclusions are still reliable by more biologically modeling CD neurons, such as synaptic nature [42] or synaptic facilitation parameters [43].

According to the domains shown in Figure 3 and 5, where different pairs of 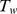 and 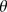 have the highest precision and reliability, it is found that a single neuron can justify its essential states due to the environment to perform information processing with high efficiency. The relationships between the input and output trains are illustrated in Figure 6, 7, and 8. The outputs of the network follow the inputs closely, which indicates that the response timing of the CD neuron is much more accurate when the neuron is in the proper states.

In our model, we did not consider the time course of the excitatory postsynaptic potential, with rise and decay times that have a strong influence on the spike time precision and reliability [53]. We only considered the sub-threshold membrane voltage because the generation of presynaptic inputs of a single neuron is not included in the intrinsic part of the IF model. Furthermore, it is valuable to attach the linear and nonlinear mechanisms in the dendrite tree in the model with respect to the properties of a real neuron and its performance. In such realistic models, it is very hard to suitably address the corresponding nonlinear dynamic behavior and the following large number of parameters. Therefore, in this paper, we mainly concentrated solely on the function of the CD neuron in the uncoupled network.

## References

[1] Shadlen MN, Newsome WT (1994) Noise, neural codes and cortical organization. Curr Opin Neurobiol 4: 569–579. 7812147 10.1016/0959-4388(94)90059-0

[2] König P, Engel AK, Singer W (1996) Integrator or coincidence detector? the role of the cortical neuron revisited. Trends Neurosci 19: 130–137. 8658595 10.1016/s0166-2236(96)80019-1

[3] Shadlen MN, Newsome WT (1998) The variable discharge of cortical neurons: implications for connectivity, computation, and information coding. J Neurosci 18: 3870–3896. 9570816 10.1523/JNEUROSCI.18-10-03870.1998 PMC6793166

[4] deCharms RC, Zador A (2000) Neural representation and the cortical code. Annu Rev Neurosci 23: 613–647. 10845077 10.1146/annurev.neuro.23.1.613

[5] Roy SA, Alloway KD (2001) Coincidence detection or temporal integration? what the neurons in somatosensory cortex are doing. J Neurosci 21: 2462–2473. 11264320 10.1523/JNEUROSCI.21-07-02462.2001 PMC6762384

[6] Rudolph M, Destexhe A (2003) Tuning neocortical pyramidal neurons between integrators and coincidence detectors. J Comput Neurosci 14: 239–251. 12766426 10.1023/a:1023245625896

[7] Sherrington C (1923) The integrative action of the nervous system. CUP Archive.

[8] Softky WR, Koch C (1993) The highly irregular firing of cortical cells is inconsistent with temporal integration of random EPSPs. J Neurosci 13: 334–350. 8423479 10.1523/JNEUROSCI.13-01-00334.1993 PMC6576320

[9] Alonso JM, Usrey WM, Reid RC (1996) Precisely correlated firing in cells of the lateral geniculate nucleus. Nature 383: 815–819. 8893005 10.1038/383815a0

[10] Stevens CF, Zador AM (1998) Input synchrony and the irregular firing of cortical neurons. Nat Neurosci 1: 210–217. 10195145 10.1038/659

[11] Salinas E, Sejnowski TJ (2000) Impact of correlated synaptic input on output firing rate and variability in simple neuronal models. J Neurosci 20: 6193–6209. 10934269 10.1523/JNEUROSCI.20-16-06193.2000 PMC6772574

[12] Usrey WM, Alonso JM, Reid RC (2000) Synaptic interactions between thalamic inputs to simple cells in cat visual cortex. J Neurosci 20: 5461–5467. 10884329 10.1523/JNEUROSCI.20-14-05461.2000 PMC6772311

[13] Agmon-Snir H, Carr CE, Rinzel J (1998) The role of dendrites in auditory coincidence detection. Nature 393: 268–272. 9607764 10.1038/30505

[14] Joris PX, Smith PH, Yin TC (1998) Coincidence detection in the auditory system: 50 years after jeffress. Neuron 21: 1235–1238. 9883717 10.1016/s0896-6273(00)80643-1

[15] Oertel D, Bal R, Gardner SM, Smith PH, Joris PX (2000) Detection of synchrony in the activity of auditory nerve fibers by octopus cells of the mammalian cochlear nucleus. Proc Natl Acad Sci U S A 97: 11773–11779. 11050208 10.1073/pnas.97.22.11773 PMC34348

[16] Marsalek P, Lansky P (2005) Proposed mechanisms for coincidence detection in the auditory brainstem. Biol Cybern 92: 445–451. 15915356 10.1007/s00422-005-0571-1

[17] Katz Y, Kath WL, Spruston N, Hasselmo ME (2007) Coincidence detection of place and temporal context in a network model of spiking hippocampal neurons. PLoS Comput Biol 3: e234. 18085816 10.1371/journal.pcbi.0030234 PMC2134961

[18] Abeles M (1982) Role of the cortical neuron: integrator or coincidence detector? Isr J Med Sci 18: 83–92. 6279540

[19] Bernander O, Douglas RJ, Martin KA, Koch C (1991) Synaptic background activity inuences spatiotemporal integration in single pyramidal cells. Proc Natl Acad Sci U S A 88: 11569–11573. 1763072 10.1073/pnas.88.24.11569 PMC53177

[20] Maršálek P (2000) Coincidence detection in the Hodgkin-Huxley equations. Biosystems 58: 83–91. 11164634 10.1016/s0303-2647(00)00110-6

[21] Krips R, Furst M (2009) Stochastic properties of coincidence-detector neural cells. Neural Comput 21: 2524–2553. 19548801 10.1162/neco.2009.07-07-563

[22] Chen Y, Yu L, Qin SM (2008) Detection of subthreshold pulses in neurons with channel noise. Phys Rev E 78: 051909. 10.1103/PhysRevE.78.051909 19113157

[23] Wang HP, Spencer D, Fellous JM, Sejnowski TJ (2010) Synchrony of thalamocortical inputs maximizes cortical reliability. Science 328: 106–109. 20360111 10.1126/science.1183108 PMC2859205

[24] Kara P, Reinagel P, Reid RC (2000) Low response variability in simultaneously recorded retinal, thalamic, and cortical neurons. Neuron 27: 635–646. 11055444 10.1016/s0896-6273(00)00072-6

[25] Riehle A, Grün S, Diesmann M, Aertsen A (1997) Spike synchronization and rate modulation differentially involved in motor cortical function. Science 278: 1950–1953. 9395398 10.1126/science.278.5345.1950

[26] Diesmann M, Gewaltig M, Aertsen A (1999) Stable propagation of synchronous spiking in cortical neural networks. Nature 402: 529–533. 10591212 10.1038/990101

[27] Diesmann M, Gewaltig M, Rotter S, Aertsen A (2001) State space analysis of synchronous spiking in cortical neural networks. Neurocomputing 38-40: 565–571.

[28] Burkitt AN (2006) A review of the integrate-and-fire neuron model: I. homogeneous synaptic input. Biol Cybern 95: 1–19. 16622699 10.1007/s00422-006-0068-6

[29] Burkitt AN (2006) A review of the integrate-and-fire neuron model: II. inhomogeneous synaptic input and network properties. Biol Cybern 95: 97–112. 16821035 10.1007/s00422-006-0082-8

[30] Herrmann A, Gerstner W (2001) Noise and the PSTH response to current transients: I. general theory and application to the integrate-and-neuron. J Comput Neurosci 11: 135–151. 11717530 10.1023/a:1012841516004

[31] Herrmann A, Gerstner W (2002) Noise and the PSTH response to current transients: II. integrateand-fire model with slow recovery and application to motoneuron data. J Comput Neurosci 12: 83–95. 12053155 10.1023/a:1015739523224

[32] Abeles M (1991) Corticonics: Neural Circuits of the Cerebral Cortex. Cambridge University Press.

[33] Carr CE, Friedman MA (1999) Evolution of time coding systems. Neural Comput 11: 1–20. 9950719 10.1162/089976699300016773

[34] Tollin DJ, Yin TCT (2002) The coding of spatial location by single units in the lateral superior olive of the cat. i. spatial receptive fields in azimuth. J Neurosci 22: 1454–1467. 11850472 10.1523/JNEUROSCI.22-04-01454.2002 PMC6757576

[35] McAlpine D, Grothe B (2003) Sound localization and delay lines–do mammals fit the model? Trends Neurosci 26: 347–350. 12850430 10.1016/S0166-2236(03)00140-1

[36] Grothe B (2003) New roles for synaptic inhibition in sound localization. Nat Rev Neurosci 4: 540–550. 12838329 10.1038/nrn1136

[37] Kandler K (2004) Activity-dependent organization of inhibitory circuits: lessons from the auditory system. Curr Opin Neurobiol 14: 96–104. 15018944 10.1016/j.conb.2004.01.017

[38] Joris P, Yin TCT (2007) A matter of time: internal delays in binaural processing. Trends Neurosci 30: 70–78. 17188761 10.1016/j.tins.2006.12.004

[39] Grande LA, Kinney GA, Miracle GL, Spain WJ (2004) Dynamic inuences on coincidence detection in neocortical pyramidal neurons. J Neurosci 24: 1839–1851. 14985424 10.1523/JNEUROSCI.3500-03.2004 PMC6730395

[40] Bender VA, Bender KJ, Brasier DJ, Feldman DE (2006) Two coincidence detectors for spike timingdependent plasticity in somatosensory cortex. J Neurosci 26: 4166–4177. 16624937 10.1523/JNEUROSCI.0176-06.2006 PMC3071735

[41] Kempter R, Gerstner W, van Hemmen JL, Wagner H (1998) Extracting oscillations. neuronal coincidence detection with noisy periodic spike input. Neural Comput 10: 1987–2017. 9804669 10.1162/089976698300016945

[42] Pantic L, Torres JJ, Kappen HJ (2003) Coincidence detection with dynamic synapses. Network: Comput Neural Syst 14: 17–33. 10.1088/0954-898x/14/1/302 12613550

[43] Mejías JF, Torres JJ (2008) The role of synaptic facilitation in spike coincidence detection. J Comput Neurosci 24: 222–234. 17674172 10.1007/s10827-007-0052-8

[44] Kostal L, Marsalek P (2010) Neuronal jitter: can we measure the spike timing dispersion differently? Chin J Physiol 53: 454–464. 21793358 10.4077/cjp.2010.amm031

[45] Kreuz T, Haas JS, Morelli A, Abarbanel HD, Politi A (2007) Measuring spike train synchrony. J Neurosci Methods 165: 151–161. 17628690 10.1016/j.jneumeth.2007.05.031

[46] Mainen ZF, Sejnowski TJ (1995) Reliability of spike timing in neocortical neurons. Science 268: 1503–1506. 7770778 10.1126/science.7770778

[47] Berry MJ, Meister M (1998) Refractoriness and neural precision. J Neurosci 18: 2200–2211. 9482804 10.1523/JNEUROSCI.18-06-02200.1998 PMC6792934

[48] Miller MI, Mark KE (1992) A statistical study of cochlear nerve discharge patterns in response to complex speech stimuli. J Acoust Soc Am 92: 202–209. 1324958 10.1121/1.404284

[49] DeWeese MR, Wehr M, Zador AM (2003) Binary spiking in auditory cortex. J Neurosci 23: 7940–7949. 12944525 10.1523/JNEUROSCI.23-21-07940.2003 PMC6740590

[50] Engel AK, Singer W (2001) Temporal binding and the neural correlates of sensory awareness. Trends Cogn Sci 5: 16–25. 11164732 10.1016/s1364-6613(00)01568-0

[51] de la Rocha J, Doiron B, Shea-Brown E, Josić K, Reyes A (2007) Correlation between neural spike trains increases with firing rate. Nature 448: 802–806. 17700699 10.1038/nature06028

[52] Usrey WM, Reid RC (1999) Synchronous activity in the visual system. Annu Rev Physiol 61: 435–456. 10099696 10.1146/annurev.physiol.61.1.435

[53] Rodriguez-Molina VM, Aertsen A, Heck DH (2007) Spike timing and reliability in cortical pyramidal neurons: effects of EPSC kinetics, input synchronization and background noise on spike timing. PLoS ONE 2: e319. 17389910 10.1371/journal.pone.0000319 PMC1828624

